# Yeast-Based Screen to Identify Natural Compounds with a Potential Therapeutic Effect in Hailey-Hailey Disease

**DOI:** 10.3390/ijms19061814

**Published:** 2018-06-20

**Authors:** Graziella Ficociello, Azzurra Zonfrilli, Samantha Cialfi, Claudio Talora, Daniela Uccelletti

**Affiliations:** 1 Department of Biology and Biotechnology “C. Darwin”, Sapienza University of Rome, 00185 Rome, Italy; graaziella.ficociello@uniorma1.it; 2 Department of Molecular Medicine, Sapienza University of Rome, 00161 Rome, Italy; azzurra.zonfrilli@uniroma1.it (A.Z.); samantha.cialfi@uniroma1.it (S.C.)

**Keywords:** Hailey-Hailey, NRF2, NOTCH1, oxidative-stress

## Abstract

The term orthodisease defines human disorders in which the pathogenic gene has orthologs in model organism genomes. Yeasts have been instrumental for gaining insights into the molecular basis of many human disorders, particularly those resulting from impaired cellular metabolism. We and others have used yeasts as a model system to study the molecular basis of Hailey-Hailey disease (HHD), a human blistering skin disorder caused by haploinsufficiency of the gene *ATP2C1* the orthologous of the yeast gene *PMR1*. We observed that *K. lactis* cells defective for *PMR1* gene share several biological similarities with HHD derived keratinocytes. Based on the conservation of ATP2C1/PMR1 function from yeast to human, here we used a yeast-based assay to screen for molecules able to influence the pleiotropy associated with *PMR1* deletion. We identified six compounds, Kaempferol, Indirubin, Lappaconite, Cyclocytidine, Azomycin and Nalidixic Acid that induced different major shape phenotypes in *K. lactis*. These include mitochondrial and the cell-wall morphology-related phenotypes. Interestingly, a secondary assay in mammalian cells confirmed activity for Kaempferol. Indeed, this compound was also active on human keratinocytes depleted of ATP2C1 function by siRNA-treatment used as an in-vitro model of HHD. We found that Kaempferol was a potent NRF2 regulator, strongly inducing its expression and its downstream target *NQO1*. In addition, Kaempferol could decrease oxidative stress of ATP2C1 defective keratinocytes, characterized by reduced NRF2-expression. Our results indicated that the activation of these pathways might provide protection to the HHD-skin cells. As oxidative stress plays pivotal roles in promoting the skin lesions of Hailey-Hailey, the NRF2 pathway could be a viable therapeutic target for HHD.

## 1. Introduction

Hailey-Hailey disease (HHD), also called benign familial pemphigus, is an autosomal dominant blistering skin disorder, manifesting in the 3rd to 4th decades of life. The overall incidence and prevalence of HHD is unknown, although some authors have reported an incidence between 1:40,000 and 1:50,000 [1,2]. The genetics and pathophysiology of this skin disorder have been linked to mutations in the *ATP2C1* gene [3,4]. The gene, located on the long arm of chromosome 3, 3q21-q24 region, encodes the human secretory pathway Ca^2+^/Mn^2+^ ATPase, hSPCA1 [5]. Although ATP2C1 is mostly localized to the Golgi apparatus, it regulates also endoplasmic reticulum (ER) Ca^2+^ stores with effects on both Golgi and ER functions. The lack of ATP2C1 in keratinocytes leads to the loss of cell-to-cell adhesion (acantholysis) among the cells of the suprabasal layer of epidermis probably due to a retraction of keratin intermediate filaments from the desmosomal plaques [6]. Although *ATP2C1* mutations are 100% penetrant, currently there is no treatment known to be effective in reducing the cutaneous manifestations of HHD. The Standard of Care (SOC) treatment consists in either topical or oral administration of corticosteroids often used in combination with topical/systemic antimicrobial agents. However, prolonged treatment course of steroids is limited due to their side effects, most commonly skin atrophy. This last aspect must be carefully considered, because in HHD-patients, lesion development is associated with the simple friction of the skin, and we found that HHD-keratinocytes are characterized by wound defects [7]. Additionally, patients develop lesions refractory to corticosteroids. As lesions became recalcitrant to SOC treatment, several possible treatments have been proposed, including: Botulinum toxin injection and photodynamic therapy [8]. However, evidence for the above indicated treatments of HHD is limited to case reports, case series, and expert opinion. The development of causal treatment strategies (i.e., molecular therapy-based) is highly desirable and could be reached through intensified efforts to elucidate the various molecular mechanisms underlying the disorder. HHD is associated with the loss of a single copy of the *ATP2C1* gene. *ATP2C1* is likely essential in humans, as more severe phenotypes are found in patients who suffer clonal loss of both copies of the gene [9]. Consistently, mice embryos homozygous for null mutations in *ATP2C1* die with defects in neural tube closure, while heterozygotes show susceptibility to squamous cell tumors, a phenotype observed rarely in humans with Hailey-Hailey; [10,11] and our personal observation); however, this mouse model fails to reproduce the clinical manifestation of the disease, unfortunately opposing the applicability of this mouse model in HHD. Yeast has been increasingly used as a model and tool for biomedical research [12,13], based on the observation that basic cellular functions are conserved from yeast to humans and that disease’s key players are often evolutionarily conserved. Indeed, about 30% of the genes known to be involved in human diseases have a yeast ortholog [14,15]. For these reasons, this simple organism is widely used for high-throughput genetic and small-molecule screens to find possible pharmacological drugs for many human diseases. This is still true in the study of Hailey-Hailey disease. Indeed, both the budding yeasts *Saccharomyces cerevisiae* (*S. cerevisiae*) and *Kluyveromyces lactis* (*K. lactis*) express the orthologous gene of *ATP2C1*, *PMR1* (plasma membrane ATPase related) [16,17,18]. Yeast cells deprived of *PMR1* display pleiotropic phenotypes; some of them have been reported also for HHD keratinocytes, including alterations in Ca^2+^ homeostasis, mitochondrial dysfunctions and an increased production of reactive oxygen species (ROS) [3,19,20]. Oxidative stress represents a hallmark of the keratinocytes derived from the lesions of HHD patients and it could be associated to the decreased action of some detoxifying systems. Particularly, we previously demonstrated that one of the detoxifying enzymes involved in the pathophysiology of HHD is the Glutathione S- transferase (GST) [21]. Indeed, performing a genetic screening, we found that the expression of mammalian GST in the yeast *K. lactis* lacking *PMR1* recovers the oxidative alterations of mutant cells, promoting a reduction to the sensitivity to ROS generating compound (H_2_O_2_), decreasing its cellular content and restoring the mitochondrial function. Additionally, we showed that, both in yeast cells and in the lesional-derived keratinocytes of HHD patients, the expression of this detoxifying gene is down-regulated [21]. Based on these observations, in this study we establish a yeast-based screening assay, designed to identify drugs that could be active against Hailey-Hailey disorder. Natural product collections are bioactive and structurally diverse molecules. It has been estimated that 60% of current FDA-approved drugs have origins in natural products, illustrating the power of these compounds in drug discovery [22]. Thus, we took advantage of a library of 131 natural compounds to analyze their ability to suppress the phenotypes of *K. lactis pmr1∆* cells. Due to the great relevance of the oxidative stress in HHD-derived keratinocytes, in the initial screening system we evaluated if the drugs were able to recover the oxidative-stress alterations of our mutant. With this aim, we analyzed the growth in the presence of H_2_O_2_ or menadione, two generators of ROS at extracellular and mitochondrial level, respectively. From the first screening, we selected six compounds that were utilized for further analysis. Specifically, we analyzed if the six positive hits were able to alleviate other main defects of *pmr1∆* cells, like the calcium homeostasis alteration, the cell wall defects and the mitochondrial dysfunction. Moreover, we showed that one of the identified hit in the yeast screening was effective also in cellular culture of keratinocytes silenced for ATP2C1. These results validate our approach that provides the use of the yeast *K. lactis* to screen drugs with potential to treat the HHD disease.

## 2. Results

### 2.1. Primary Screen of Chemical Libraries Using KLPMR1-Based Assay

**Figure 1:**
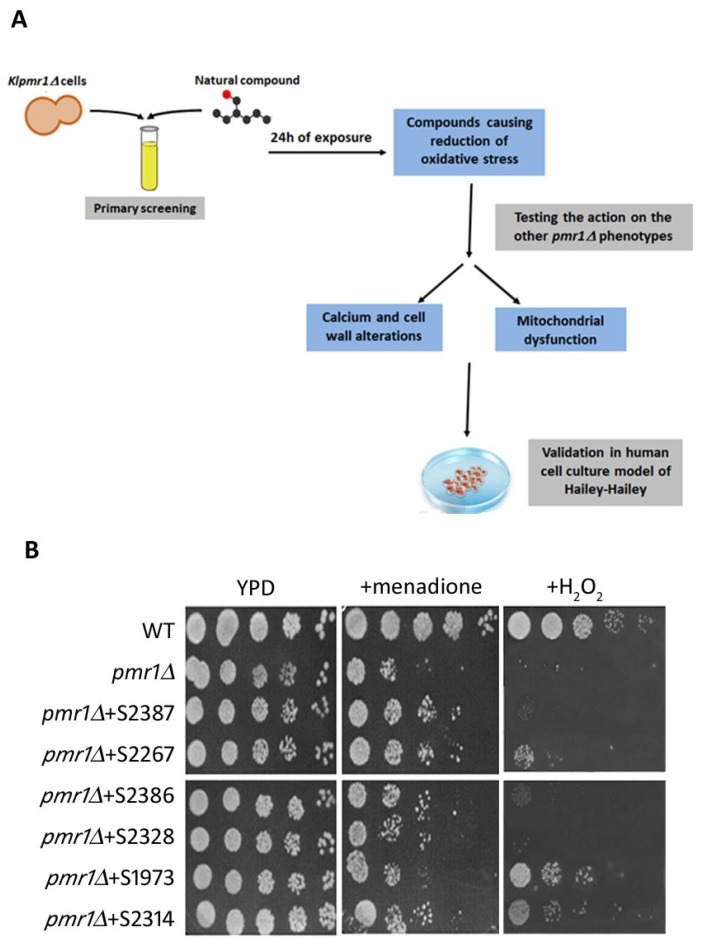
(**A**) General flowchart of the natural products screen approach. The primary screening is performed to identify compounds that alleviate the oxidative stress of *pmr1*-mutant cells. Then the effects of the positive hits are tested for the other phenotypes of the mutant strain. The final step is to test the selected molecules on the human cell cultures used as model for Hailey-Hailey disease; (**B**) The *PMR1*-deleted strain exposed or not for 24 h to different natural products was tested for its ability to grow with or without the 60 µM menadione or 4 mM H_2_O_2_. Wild type cells (WT) were used as control.

Previously, we demonstrated the feasibility of using the yeast Kluyveromyces lactis for modeling Hailey-Hailey disease [21]. In the present study we performed a pharmacological screening using a library of 131 natural molecules to analyze their ability to suppress the *pmr1∆* phenotypes (Figure 1A). The drug collection includes inhibitors, activators and antagonists acting on molecular targets involved in different signaling pathways. Since the lack of the Golgi Ca^2+^-ATPase in yeast, as well as in human HHD keratinocytes, induced a prominent increase of ROS production [20,23], we started our screening testing with the natural molecules capability to ameliorate the growth properties of the *pmr1∆* strain under oxidative-stress conditions. Toward this aim, ROS conditions were achieved either exogenously by H_2_O_2_ administration or endogenously by menadione treatment (Figure 1B). First, we tested all the molecules at a concentration of 200 M. The compounds that had partial or no effects were tested at a concentration of 250 M. Furthermore, the molecules found to be toxic at 200 M were analyzed at a lower concentration (100, 10 and 5 M) and those compounds showing either toxicity or ineffectiveness at a lower concentration were excluded from further analysis (Table S1). As shown in Figure 1B, six compounds were able to reduce the sensitivity of *klpmr1∆* mutant to menadione and/or H_2_O_2_. The molecules S2328, S2386, S2314 and, more effectively, the S2387 and S2267 decreased the growth defects of mutant cells in the presence of menadione. Meanwhile, S1973 and S2314 were more effective against H_2_O_2_ (Figure 1B). This indicates that the action of the different compounds depends on the localization of the ROS source. These six molecules, selected from the preliminary screen, belong to different class of drugs. Indeed, the S2386 and S1973 (Indirubin and Cyclocytidine) are used in medicine as chemotherapeutics [24,25], S2387 and S2314 (Lappaconite Hydrobromide and Kaempferol) have an anti-inflammatory action [26], and S2328 and S2267 (Nalidixic acid and Azomycin) are mainly recognized as anti-bacterial drugs [27]. A dose–response curve was then performed for the six compounds (Figure S1) and the EC50 was determined as reported in Table 1. 

### 2.2. Yeast-Hits Rescue Multiple Defects in pmr1Δ Cells 

**Figure 2:**
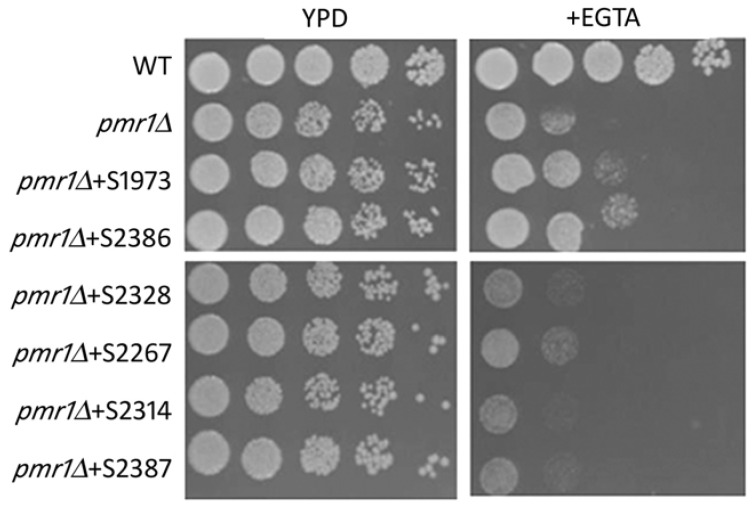
Analysis of calcium alteration. WT and *Klpmr1∆* cells exposed or not to the individual natural molecules, were grown for 24 h in Yeast Extract-Peptone-Dextrose (YPD) medium at 30 °C. Then, serial dilutions of the cultures were spotted onto solid medium supplemented or not with 20 mM EGTA. Scale bar:

Our next goal was to assess if the hits selected in our screening were also able to rescue the multiple phenotypes associated with the deletion of the *PMR1* gene. As reported by [20], the deletion of the *PMR1* in *K. lactis* cells led to a higher content of intracellular calcium as well as growth defects when the homeostasis of this ion is disrupted by EGTA. For this aim, we tested the sensitivity of the *Klpmr1∆* cells treated with the selected hits to the calcium chelator EGTA. As indicated in Figure 2, only two compounds had a positive effect: S2386 and S1973, meanwhile the other four molecules were ineffective. 

### 2.3. Cell Wall Phenotype

**Figure 3:**
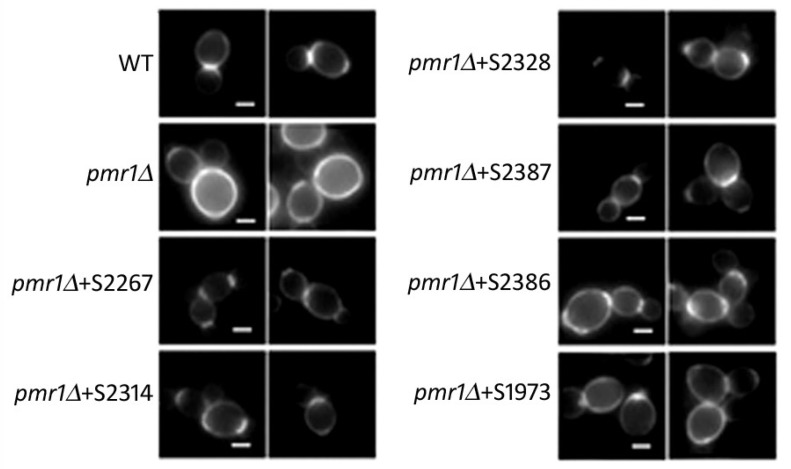
Chitin distribution of mutant cells treated with the six selected products. *PMR1*-deleted cells, grown with or without the individual compounds for 24 h at 30 °C, were stained with the chitin-binding dye CFW. At least 500 cells were analyzed for each treatment to determine the percentage of cell wall recovery. Wild type cells (WT) represent the positive control. Scale bar 2 µm.

*K. lactis* strain deleted for *PMR1* gene had defects in the cell wall organization. Indeed, as shown by using the fluorescent dye Calcofluor white (CFW), which binds the cell wall component chitin, we previously reported that in wild type cells the chitin is mainly deposited to the bud-emergence sites, whereas in the *Klpmr1∆* strain the fluorescence is distributed across the entire cell wall [17]. To analyze the effect of the selected hits on the cell wall structure of our mutant, the CFW staining was performed. We found that all the six molecules were able to recover the wild type-like chitin distribution (Figure 3). However, the molecules S1973 and S2314 induced a recovery of 40%; meanwhile, the compounds S2267, S2387 and S2386 relieved the wall disorganization in about 50% of the cells. Particularly, the compound S2328 strongly recovered the cell wall morphology in 80% of *klpmr1∆* cells.

### 2.4. Mitochondrial Morphology

**Figure 4:**
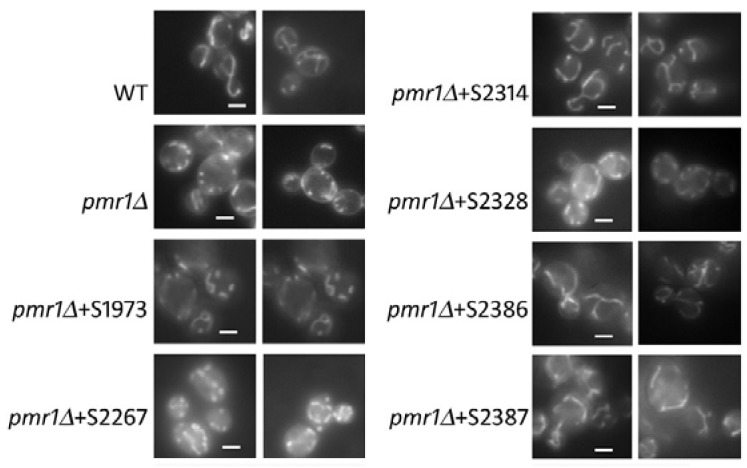
Effect of the natural compounds on the altered mitochondrial function of *pmr1∆* cells. The mutant cells, untreated or treated with the indicated molecules for 24 h, were stained with the vital dye DASPMI and immediately the fluorescence micrographs were taken. To calculate the percentage of cells with altered tubular mitochondria morphology, at least 500 cells were analyzed for each condition. Wild type strain (WT) was used as a control. Scale bar 2 µm.

Mitochondria are responsible for the main source of ROS in most cells, linking mitochondrial respiration with ROS effects on cellular function [28]. Wild type cells show a tubular network of mitochondria as long as in the *Klpmr1∆* strain these organelles appear as dots, indicating an alteration in their functionality [20]. Thus, we addressed the capability of the selected hits to rescue the mitochondria alteration of *Klpmr1∆* cells. With this aim, we incubated our sample with the fluorescent probe DASPMI that is taken up by mitochondria as a function of membrane electrochemical potential. As shown in Figure 4, we found that three drugs (S2386, S2314 and S2387) restored the wild type-like tubular network of mitochondria. The drug S2314 totally relieved the mitochondria defects of our mutant while the molecules S2386 and S2387 worked in the 60% of *Klpmr1∆* cells. Overall, our data indicate that each molecule acts on specific phenotypes of *Klpmr1∆* cells (Table S2). This is in agreement with the fact that the selected molecules belong to different classes of drugs.

### 2.5. Drugs Active in the Yeast-Based Assay Were Also Active in Human Cells

**Figure 5:**
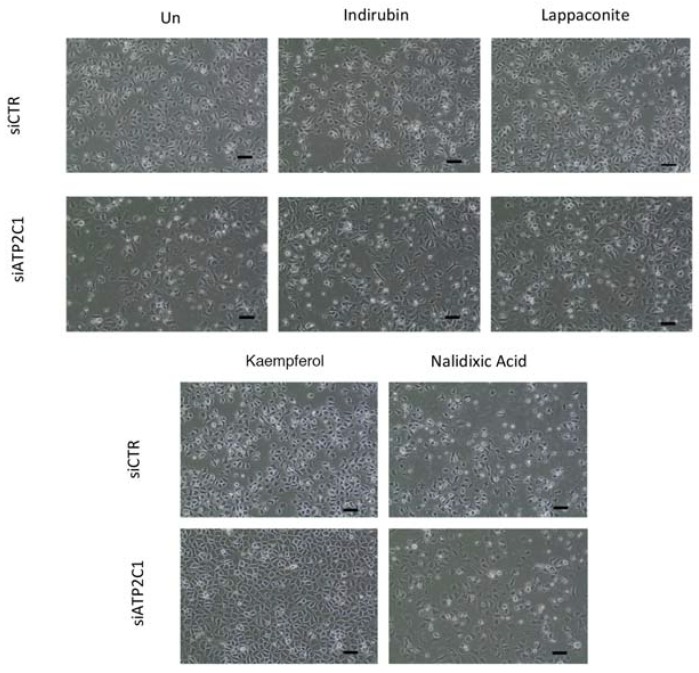
Keratinocytes-derived cell line, HaCaT, was transfected with either siRNA-CTR or siRNA-ATP2C1; 24 h post-transfection, cells were treated with the indicated compounds at 10 µM for a further 24 h and analyzed by microscopy. (100× magnification). The potencies (EC_50_ = 0.8 µM +/− 0.1) of Kaempferol were obtained from the dose–response curves using GraphPad Prism (GraphPad Software, La Jolla, CA, USA). Scale bar: 50 µm.

**Figure 6:**
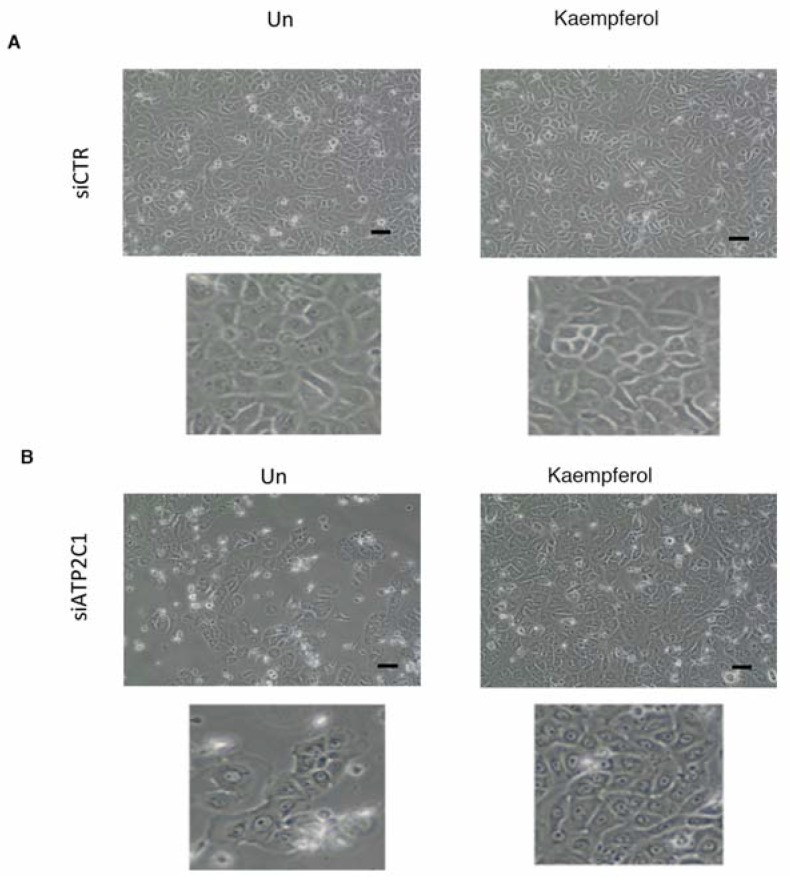
(**A**,**B**) NHKCs (primary human keratinocytes) were transfected with control (siRNA-CTR) or ATP2C1-specific siRNA oligonucleotides; 24 h later, cells were treated with Kaempferol (10 µΜ) for 24 h and analyzed by microscopy. (100× magnification). Each of the lower images is an enlarged subset of the image above. Scale bar: 50 µm.

We next tested the compounds that were active in our yeast-based assay in a human cell-based model of HHD-disease; in particular, we used a siRNA-ATP2C1 to mimic *ATP2C1*-loss of function [7,23,29]. We previously found that siATP2C1-treated cells share most of the defects observed in *K. lactis* cells defective for *PMR1* gene including oxidative stress [7,21,23,29,30]. We established that ATP2C1 inhibition in both immortalized and primary keratinocyte cells results in an impaired proliferation; thus, the compounds from the yeast screening were tested in human keratinocytes and we sought to perform our primary screen in HaCaT cells determining how the morphology/cell proliferation defects of si-ATP2C1 cells were influenced by the treatment with the identified compounds. HaCaT cells were transfected with either siATP2C1 or -siCTR after 24 h treated with the indicated compound for further 24 h. Interestingly, we observed that Kaempferol (EC50 0.8 µM) treatment rescued the aberrant cell morphology and cell growth ability of siATP2C1-treated cells (Figure 5). In a second step, we validated the Kaempferol effect by performing a secondary analysis in human primary keratinocytes. Similarly, to our observation in HaCaT cells, we observed that Kaempferol treatment was able to rescue the aberrant cell morphology/growth of siATP2C1 treated human primary keratinocytes (Figure 6).

### 2.6. Potential Mechanism of Kaempferol against ATP2C1-Induced Oxidative-Stress through Regulation of Nuclear Factor Erythroid-2-Related Factor 2 Signaling

**Figure 7:**
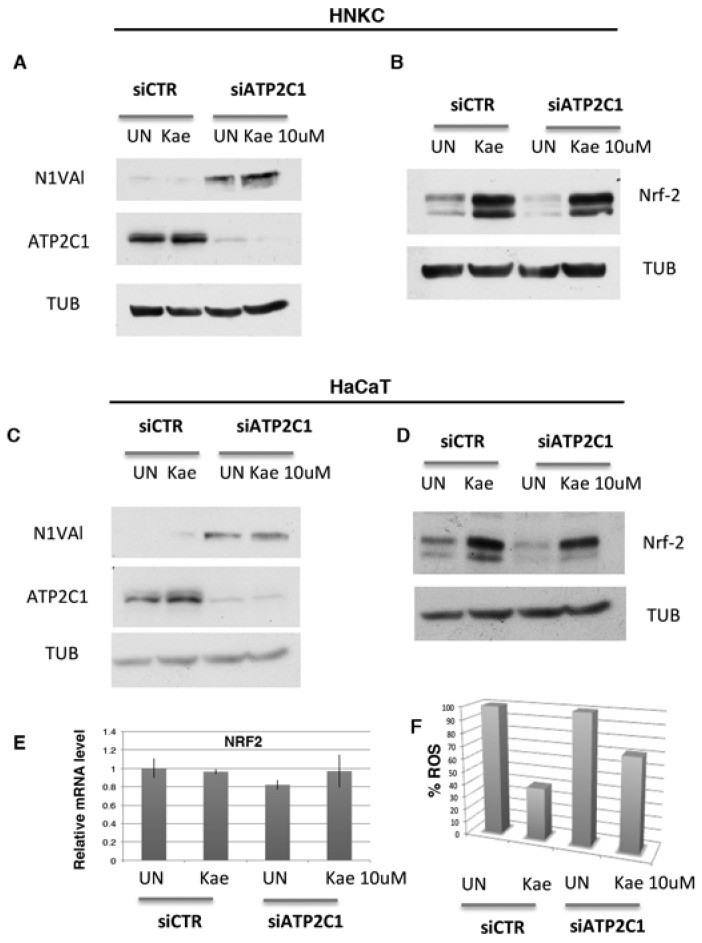
Cell extracts were prepared from both NHKCs (**A**,**B**) and HaCaT cells (**C**,**D**) transfected with either control (siRNA-CTR) or ATP2C1-specific siRNA oligonucleotides; 24 h later, cells were treated with Kaempferol (10 µΜ) for 24 h and the cell extracts analyzed by western blot; (**E**) Cells were treated as in C, and expression of NRF2 was determined by RT-PCR; (**F**) Keratinocytes-derived cell line, HaCaT, was transfected with either siRNA-CTR or siRNA-ATP2C1 and cells were analyzed by flow cytometry. The percentage of ROS-positive cells is also shown. The absolute value of ROS of both from siRNA-CTR and siRNA-ATP2C1 Kaempferol-untreated cells was arbitrary indicated as 100%.

**Figure 8:**
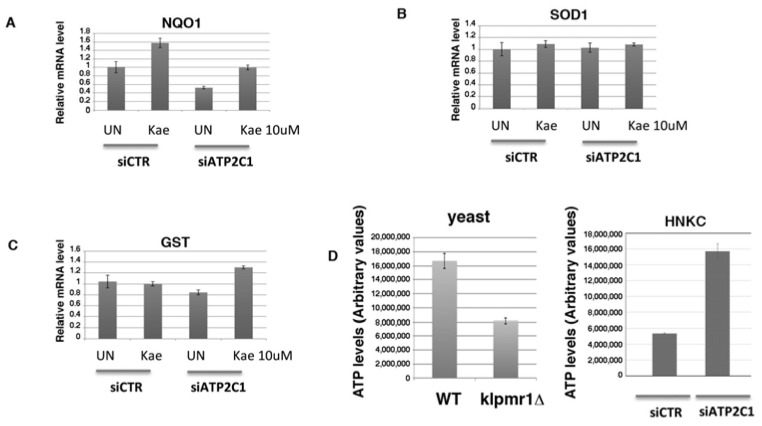
(**A**–**C**) NHKC cells were transfected with control (siRNA-CTR) or ATP2C1-specific siRNA oligonucleotides; 24 h later, cells were treated with Kaempferol (10 µΜ) for 24 h, the total RNA extracted, and the expression of the indicated targets analyzed by RT-PCR; (**D**) ATP production in both yeast (left) and primary human keratinocytes (right). ATP levels were analyzed in *Klpmr1∆ and WT* cells, and ATP production was assessed in primary human keratinocytes transfected with control (siRNA-CTR) or ATP2C1-specific siRNA oligonucleotides.

HHD lesion-derived keratinocytes are characterized by increased oxidative stress and decreased expression levels of both NOTCH1 and NRF2 [7]. We have established that altered function of these factors plays an important role in the alteration observed in HHD-derived keratinocytes [7]. Both NOTCH1 and NRF2 factors are important determinant of skin homeostasis and we found that they are differentially regulated between normal and HHD-derived keratinocytes, as well as in HaCaT cells interfered for ATP2C1 function [7]. Thus, we tested the expression of these two factors in response to Kaempferol treatment. Both HaCaT cells and primary human keratinocytes were transfected with either siATP2C1 or -siCTR, and 24 h post-transfection, they were treated with Kaempferol for a further 24 h. In both cell types NOTCH1 expression wasn’t affect by Kaempferol treatment, while NRF2 expression was strongly increased. This observation indicates that loss of NRF2 activity in defective *ATP2C1*-cells might have a direct effect on increased oxidative-stress (Figure 7). Thus, the antioxidant property of Kaempferol and its ability to restore NRF2 expression might play a role in reducing the oxidative-stress of siATP2C1-treated cells. Interestingly, Kaempferol did not significantly affect the steady-state level of *NRF2* mRNA, indicating that it stimulates NRF2 expression by protein stabilization (Figure 7). NRF2 activation directly regulates antioxidant gene transcription [31,32]. NRF2 activation can be modulated by flavonoid as Kaempferol [31,32]; thus, the reduced expression NRF2 in lesioned HHD skin may play a role in the transcriptional down-regulation of antioxidant genes [7,21,30]. Therefore, we first tested if Kaempferol treatment affects the level of oxidative-stress present in siATP2C1-treated cells (Figure 7F). The percentage of DFCA-positive cells in siATP2C1 cells reached 40–60% at 48 h after transfection, whereas only 15% of the siRNA-CTR control cells were DFCA-positive (data not shown and [7]). Interestingly, treatment with Kaempeferol reduced the oxidative stress of both siCTR and of siATP2C1-treated keratinocytes. However, the extent of oxidative-stress in siATP2C1 still remained higher than siCTR cells, indicating that the causative factors and underlying mechanism of oxidative stress still remain active (Figure 7F). NRF2 directly affects the homeostasis of ROS by regulating the expression of several antioxidant genes. Therefore, we analyzed the expression of NRF2-target genes in siATP2C1 and si-CTR-interfered primary keratinocytes after Kaempferol treatment. In ATP2C1 defective cells we observed the loss of NRF2 protein expression; however, only two down-regulated genes (*NQO1* and *GST-M1*) were similarly altered by siRNA-ATP2C1 treatment (Figure 8). Kaempferol treatment partially suppressed oxidative stress in ATP2C1-defective cells and this was paralleled by increased levels of NRF2 and *NQO1/GST-M1* expression. Our data indicate that Kaempferol treatment rescues the impaired NRF2 expression of ATP2C1 defective cells and, in turn, *NQO1/GST-M1* expression (Figure 8).

### 2.7. Increased Mitochondrial Activity as a Source of Oxidative Stress in ATP2C1-Defective Keratinocytes

Reactive oxygen species (ROS) are generated as a by-product of mitochondrial oxidative phosphorylation. However, inhibition of the mitochondrial electron transport induces generation of ROS that results in mitochondrial dysfunction [33]. NQO1 influences several aspects of mitochondrial function including: elevation of mitochondrial complex I activity; increased ATP production; maintenance of an elevated NAD/NADH ratio; and decreased ROS production ([33] and references therein). *ATP2C1* defective keratinocytes are characterized by reduced *NQO1* expression (Figure 8). Thus, we tested the ATP production in both *ATP2C1*-defective keratinocytes and *Klpmr1∆* cells as a sign of mitochondrial function; we found that ATP production was decreased in *Klpmr1∆* cells in line with the observed altered mitochondrial shape, indicating that mitochondrial dysfunction results in a cascade of events that include reduced ATP production (Figure 8D). Unexpectedly, we found that ATP production was increased in keratinocytes depleted of ATP2C1 function (Figure 8D). This observation indicates that in ATP2C1-defective mammals, cells with increased oxidative stress may be determined, at least in part, by increased mitochondrial activity, rather than by its dysfunction.

## 3. Discussion

Calcium [Ca^2+^] serves as an ubiquitous second messenger in all eukaryotes [34]. There are several biological processes regulated by temporally and spatially defined changes of Ca^2+^ concentration in the cytoplasm or in defined organelles [34]. ATPase pumps modulate global cytosolic calcium levels and/or may control only the calcium levels, in particular intracellular calcium stores, e.g., endoplasmic reticulum, Golgi. In this context, one regulator of Golgi luminal calcium levels is the secretory pathway calcium ATPase 1 (SPCA1), an active transporter of calcium into the secretory pathway [4,35,36,37,38]. Mutations in *ATP2C1* (SPCA1) manifest as Hailey-Hailey disease, an autosomal dominant skin disorder [3]. Hailey-Hailey disease is characterized mainly by skin-specific phenotype symptoms characterized by the loss of cell-cell adhesion (acantholysis) [3]. Mutations in *ATP2C1* result in the decrease of ATP2C1 protein expression. There have been very few studies addressing the consequences of ATP2C1 inhibition in mammalian cells. In this context, yeast systems have become an attractive choice for the study of functionally conserved ATP2C1 function. We have developed a model yeast system to study the poorly defined genetic functions of the *ATP2C1* gene in Hailey-Hailey disease development. Cellular phenotypes associated with *ATP2C1/PMR1* loss of function in yeast can be investigated to clarify the cellular and molecular functions of *ATP2C1* in keratinocytes. In line with the notion that Ca^2+^ signal regulates a multitude of downstream responses, we show here that the strain bearing the *KlPMR1* gene disruption exhibited a pleiotropic phenotype. The pleiotropy of the mutant suggests that Pmr1 steers different calcium-dependent signal pathways to control distinct physiological processes. Here, we carried out a phenotypic screening to identify compounds able to revert either single or multiple phenotypes of *Klpmr1∆* strain. Thus, understanding the mechanism of selective rescue by these compounds would shed light on the relevant molecular mechanisms to target for therapy. In this frame, we performed a pharmacological screening using a library of 131 natural molecules to analyze their ability to suppress the *Klpmr1∆* phenotypes. Since the lack of the Golgi Ca^2+^-ATPase in yeast, as well as in human HHD keratinocytes, induced a prominent increase of ROS production [20,23], we analyzed first the capability of the library to ameliorate the growth properties of the *Klpmr1∆* strain under oxidative stress conditions. ROS conditions were achieved either exogenously by H_2_O_2_ administration or endogenously by menadione treatment. Six compounds, Indirubin (S2386), Cyclocytidine (S1973), Lappaconite Hydrobromide (S2387), Kaempferol (S2314), Nalidixic acid (S2328) and Azomycin (S2267) were able to reduce the sensitivity of *Klpmr1∆* mutant to menadione and/or H_2_O_2_. The molecules S2328, S2386, S2314 and, more effectively, S2387 and S2267 decreased the growth defects of mutant cells in the presence of menadione. Meanwhile, S1973 and S2314 were more effective against H_2_O_2_. This indicates that the action of the different compounds depends on the localization of the ROS source. Then, we addressed if the six compounds selected in our screening were also able to rescue the multiple phenotypes associated with the deletion of the *PMR1* gene. Our findings indicated that selected drugs were able to target the phenotypic traits of *Klpmr1∆* mutant in both a multiple and phenotype-specific manner. Indeed, we found that Indirubin and Cyclocytidine, but not the other selected compounds, were able to revert the defect of *Klpmr1∆* in regulation of intracellular calcium homeostasis as displayed by alterations in the sensitivity to Ca^2+^ chelator EGTA. Conversely, all six molecules were able to recover the defective wall structure of the mutant. However, we found that different molecules exhibited different strengths of suppression, with Nalidixic acid recovering cell wall morphology in 80% of *klpmr1∆* cells. Similarly, we found that of the six selected compounds, three drugs (S2386, S2314 and S2387) restored the wild type-like tubular network of mitochondria. Among these compounds, only S2314 fully rescued the mitochondrial defects. The observation that compounds suppress different sets of phenotypes and at different strengths may suggest that Pmr1 loss of function elicits distinct and separable downstream responses. We have proven that yeast represents a useful model organism for investigating molecular and cellular aspects of Hailey-Hailey diseases, which may help to develop precise therapies for this disorder. Here we found six compounds able to ameliorate the multiple phenotypes of *klpmr1∆* cells, summarized in the Supplementary Table S2. 

Among the selected molecules, we found that Kaempferol was particularly effective for reverting the oxidative alterations of mutant cells, alleviating the sensitivity to ROS-generating compounds (H_2_O_2_ and menadione), decreasing the ROS cellular content, and restoring the mitochondrial function. Interestingly, we observed that Kaempferol treatment rescued the aberrant cell morphology and cell growth ability of siATP2C1-treated keratinocyte cells (Figure 5 and Figure 6). Moreover, NRF2 expression was increased by Kaempferol treatment, further supporting our observation that loss of NRF2 activity in defective *ATP2C1* cells may have a direct effect on increased oxidative stress. Interestingly, treatment with Kaempferol reduced the oxidative stress of si*ATP2C1*-treated keratinocytes. Moreover, Kaempferol treatment induced an increase of NRF2 expression, as well as a reduction of oxidative stress, in si-ATP2C1-treated keratinocytes. These data further support our observation that loss of NRF2 activity in defective *ATP2C1* cells may have a direct effect on increased oxidative stress. ROS are generated as a by-product of mitochondrial oxidative phosphorylation. Here we found that mitochondrial integrity is altered by inactivation of the *K. lactis PMR1* gene, and Kaempferol treatment led to the rescue of mitochondrial phenotype together with decreased sensitivity to ROS sources. Thus, we tested the mitochondrial functionality of both *klpmr1∆* cells and siATP2C1-interfered human keratinocytes by analyzing ATP production. In line with the altered mitochondrial morphology, ATP production was decreased in *Klpmr1∆* cells (Figure 8D). Unexpectedly, we found that ATP production was increased in keratinocytes depleted of ATP2C1. Stimulation of mitochondrial oxidative metabolism by Ca^2+^ is now generally recognized as an important mechanism for the control of cellular ATP homeostasis. Increases in cytosolic calcium results in an increased mitochondrial Ca^2+^ uptake and ATP synthesis [33]. Thus, it may be possible that in ATP2C1-defective mammals, the increased oxidative stress of cells is determined by increased mitochondrial activity, rather than by its dysfunction. 

## 4. Materials and Methods

### 4.1. Yeast Strains, Growth Conditions 

The strains used in this study were MW278-20C (MAT a, ade2, leu2, uraA) and CPK1 (MAT a, ade2, leu2, uraA, PMR1::KanR). The yeast growth media used for all the experiments was YPD medium (1% yeast extract, 1% peptone, 2% glucose, DIFCO (Difco, Becton Dickinson, Sparks, MD, USA)).

### 4.2. Library Screen

For the screening, a library of 131 natural products was purchased from Selleck Chemicals (Houston, TX, USA). The complete list of chemicals screened is provided in Supplementary Table S1. Compounds were stored as 10 mM stock solutions in dimethyl sulfoxide (DMSO) at −20 °C until use. Compound stocks were diluted in a volume of 1 mL of YPD to the indicated concentrations. After 24 h of growth at 28 °C, five-fold serial dilution of cultures were spotted onto YPD agar plates supplemented or not with 60 µM menadione or 4 M H_2_O_2_ or 20 mM EGTA, as indicated. The plates were incubated at 30 °C for 3 days. To determine a dose–response curve, mutant cells were treated at 30 °C for 24 h with increasing concentrations of each chemical diluted in 1 mL of YPD at the indicated concentrations. After that, cells were diluted to 0.07 OD_600_ in 2 mL of YPD containing 3 mM H_2_O_2_ or 20 µM menadione. After 24 h of growth at 30 °C the optical density (OD) was measured. The EC50 of the compounds was obtained from the dose–response curves using GraphPad Prism (GraphPad Software, La Jolla, CA, USA).

### 4.3. Fluorescence Microscopy

After 24 h of treatment with the molecules, the yeast cells grown in YPD medium were harvested, washed with water and then mixed 1:1 with the vital dye 2-(4-dimethylaminostyryl)-*N*-methylpyridinium iodide (DASPMI) as described in [39]. The chitin staining was performed using the probe Calcofluor White (CFW) by the method of [17]. Epifluorescence microscopy was carried out with a Zeiss AxioVert 25 microscope fitted with a ×100 immersion objective and a standard filter set.

### 4.4. Primary Human Keratinocytes

Primary human keratinocytes were purchased from (Thermo Fisher Scientific, Waltham, MA, USA). Cells were maintained in modified low calcium medium (EpiLife, Thermo Fisher, Waltham, MA, USA). Cells at passages 1 and 2 were used for study purposes.

### 4.5. Cell Culture and Transfection

Primary human keratinocytes and HaCaT cells (70–80% confluent) were maintained in modified low-calcium medium and transfected using the Lipofectamine -RNAiMAX transfection Reagent according to manufacturer’s instructions (Thermo Fisher Scientific, Waltham, MA, USA). Primary keratinocytes were transfected with 100 nmol·L^−1^ small interfering RNAs (siRNAs) for validated human ATP2C1 (L-006119-00; Thermo Scientific/Dharmacon, Lafayette, CO, USA) and the corresponding control scrambled siRNAs cells were analyzed at the indicated times after transfection by either CMH2DCFDA analysis for ROS detection or Western blot as indicated [23].

### 4.6. Reagents and Immunoblotting

ATP2C1 antibodies were purchased from Abcam (Cambridge, MA, USA) and NOTCH1 (N1Val) and NRF2 were purchased from Cell Signaling Technology (Beverly, MA, USA). All cell extracts were prepared according to the manufacturer’s instructions for detection of phosphor-ERK (Cell Signaling Technology, Beverly, MA, USA) as previously described [30]. Adenosine triphosphate (ATP) content of keratinocytes was determined using the luciferin reaction, ATP Determination Kit (Thermo Fisher Scientific, Waltham, MA, USA). A standard curve was made by using solutions containing increasing concentrations of ATP.

### 4.7. RNA Analysis and Reverse Transcriptase-Polymerase Chain Reaction

Total RNA was isolated from cells in guanidine isothiocyanate (Trizol reagent, Thermo Fisher Scientific, Waltham, MA, USA) and further processed by reverse transcriptase polymerase chain reaction (RT-PCR) as described in [40]. Each sample was analyzed in triplicate by qRT-PCR and in at least three independent experiments. qRT-PCR was performed at the opportune annealing temperature with the primers indicated in Table 2 with SensiFAST SyBr Hi-ROX kit (Bioline, London, UK) or with specific TaqMan MGB primers/probe using Taqman gene expression assay (Thermo Fisher Scientific, Waltham, MA, USA)

## 5. Conclusions

In this work we have explored the use of the yeast *K. lactis* as a tool to identify specific compounds that target specific cellular phenotypes and obtain more insight into mechanisms of disease pathology by probing the mechanisms involved in their action. Oxidative stress represents a hallmark of both *Kluyveromyces lactis* lacking *PMR1* and keratinocytes derived from the lesional areas of HHD patients and it could be associated with the decreased action of the transcription factor NRF2, involved in the regulation of several detoxifying factors. Our results indicated that molecules able to promote activation of this pathway might provide protection to the HHD-skin cells.

## Figures and Tables

**Table 1:** Median effective concentration (EC50) of the compounds selected in the yeast primary screening.

Chemicals	Oxidative Stress Selection	EC50 (mM)
S1973 (Cyclocytidine)	H_2_O_2_	51.26 ± 1.19
S2267 (Azomycin)	Menadione	102.64 ± 0.50
S2314 (Kaempferol)	H_2_O_2_	50.5 ± 0.44
S2328 (Nalidixic acid)	Menadione	50.13 ± 4.84
S2386 (Indirubin)	Menadione	48.56 ± 3.01
S2387 (Lappaconite)	Menadione	9.35 ± 0.42

**Table 2:** qRT-PCR primers.

**SyBr Green Assays**	**Sequence 5′-3′**
GST-M1 Fw	AGAGGAGAAGATTCGTGTGG
GST-M1 Rev	TGTTTCCTGCAAACCATGGC
GAPDH Fw	TGCACCACCAACTGCTTAG
GAPDH Rev	GAGGCAGGGATGATGTTC
**Taqman Gene Expression Assays**	**Assay Reference Number**
NFE2L2 (NRF2)	Hs00975961_g1
GAPDH	Hs99999905_m1
NQO1	Hs02512143_s1
SOD1	Hs00533490_m1

## Supplementary Materials

The following are available online at http://www.mdpi.com/1422-0067/19/6/1814/s1.

## References

[1] Kellermayer R. (2005). Hailey-Hailey disease as an orthodisease of PMR1 deficiency in Saccharomyces cerevisiae. FEBS Lett..

[2] Raiko L., Siljamäki E., Mahoney M.G., Putaala H., Suominen E., Peltonen J., Peltonen S. (2012). Hailey-Hailey disease and tight junctions: Claudins 1 and 4 are regulated by ATP2C1 gene encoding Ca^2+^/Mn^2+^ ATPase SPCA1 in cultured keratinocytes. Exp. Dermatol..

[3] Hu Z., Bonifas J.M., Beech J., Bench G., Shigihara T., Ogawa H., Ikeda S., Mauro T., Epstein E.H. (2000). Mutations in ATP2C1, encoding a calcium pump, cause Hailey-Hailey disease. Nat. Genet..

[4] Sudbrak R., Brown J., Dobson-Stone C., Carter S., Ramser J., White J., Healy E., Dissanayake M., Larrègue M., Perrussel M. (2000). Hailey-Hailey disease is caused by mutations in ATP2C1 encoding a novel Ca^2+^ pump. Hum. Mol. Genet..

[5] Wuytack F., Raeymaekers L., Missiaen L. (2003). PMR1/SPCA Ca^2+^ pumps and the role of the Golgi apparatus as a Ca^2+^ store. Pflugers Arch..

[6] Dobson-Stone C., Fairclough R., Dunne E., Brown J., Monaco A.P., Hovnanian A., Dissanayake M., Munro C.S., Strachan T., Burge S. (2002). Hailey-Hailey disease: Molecular and clinical characterization of novel mutations in the ATP2C1 gene. J. Investig. Dermatol..

[7] Cialfi S., Le Pera L., De Blasio C., Mariano G., Palermo R., Zonfrilli A., Uccelletti D., Palleschi C., Biolcati G., Barbieri L. (2016). The loss of ATP2C1 impairs the DNA damage response and induces altered skin homeostasis: Consequences for epidermal biology in Hailey-Hailey disease. Sci. Rep..

[8] Arora H., Bray F.N., Cervantes J., Falto Aizpurua L.A. (2016). Management of familial benign chronic pemphigus. Clin. Cosmet. Investig. Dermatol..

[9] Poblete-Gutiérrez P., Wiederholt T., König A., Jugert F.K., Marquardt Y., Rübben A., Merk H.F., Happle R., Frank J. (2004). Allelic loss underlies type 2 segmental Hailey-Hailey disease, providing molecular confirmation of a novel genetic concept. J. Clin. Investig..

[10] Mohr M.R., Erdag G., Shada A.L., Williams M.E., Slingluff C.L., Patterson J.W. (2011). Two patients with Hailey-Hailey disease, multiple primary melanomas, and other cancers. Arch. Dermatol..

[11] Okunade G.W., Miller M.L., Azhar M., Andringa A., Sanford L.P., Doetschman T., Prasad V., Shull G.E. (2007). Loss of the Atp2c1 secretory pathway Ca^2+^-ATPase (SPCA1) in mice causes Golgi stress, apoptosis, and midgestational death in homozygous embryos and squamous cell tumors in adult heterozygotes. J. Biol. Chem..

[12] Mager W.H., Winderickx J. (2005). Yeast as a model for medical and medicinal research. Trends Pharmacol. Sci..

[13] Perocchi F., Mancera E., Steinmetz L.M. (2008). Systematic screens for human disease genes, from yeast to human and back. Mol. Biosyst..

[14] Botstein D., Chervitz S.A., Cherry J.M. (1997). Yeast as a model organism. Science.

[15] Foury F. (1997). Human genetic diseases: A cross-talk between man and yeast. Gene.

[16] Rudolph H.K., Antebi A., Fink G.R., Buckley C.M., Dorman T.E., LeVitre J., Davidow L.S., Mao J.I., Moir D.T. (1989). The yeast secretory pathway is perturbed by mutations in PMR1, a member of a Ca^2+^ ATPase family. Cell.

[17] Uccelletti D., Mancini P., Farina F., Morrone S., Palleschi C. (1999). Inactivation of the KIPMR1 gene of Kluyveromyces lactis results in defective cell-wall morphogenesis. Microbiology.

[18] Voisset C., Garcia-Rodriguez N., Birkmire A., Blondel M., Wellinger R.E. (2014). Using yeast to model calcium-related diseases: Example of the Hailey-Hailey disease. Biochim. Biophys. Acta.

[19] Szigeti R., Kellermayer R. (2006). Autosomal-dominant calcium ATPase disorders. J. Investig. Dermatol..

[20] Uccelletti D., Farina F., Pinton P., Goffrini P., Mancini P., Talora C., Rizzuto R., Palleschi C. (2005). The Golgi Ca^2+^-ATPase KlPmr1p function is required for oxidative stress response by controlling the expression of the heat-shock element HSP60 in Kluyveromyces lactis. Mol. Biol. Cell.

[21] Ficociello G., Zanni E., Cialfi S., Aurizi C., Biolcati G., Palleschi C., Talora C., Uccelletti D. (2016). Glutathione S-transferase circle minus-subunit as a phenotypic suppressor of pmr1 Delta strain, the Kluyveromyces lactis model for Hailey-Hailey disease. BBA Mol. Cell Res..

[22] Newman D.J., Cragg G.M. (2007). Natural products as sources of new drugs over the last 25 years. J. Nat. Prod..

[23] Cialfi S., Oliviero C., Ceccarelli S., Marchese C., Barbieri L., Biolcati G., Uccelletti D., Palleschi C., Barboni L., De Bernardo C. (2010). Complex multipathways alterations and oxidative stress are associated with Hailey-Hailey disease. Br. J. Dermatol..

[24] Damiens E., Baratte B., Marie D., Eisenbrand G., Meijer L. (2001). Anti-mitotic properties of indirubin-3′-monoxime, a CDK/GSK-3 inhibitor: Induction of endoreplication following prophase arrest. Oncogene.

[25] Novotny L., Reichelova V., Balazova E., Ujhazy V. (1990). Comparison of some biochemical parameters of arabinosylcytosine and cyclocytidine in L1210 murine leukemia cells. Neoplasma.

[26] Murota K., Shimizu S., Miyamoto S., Izumi T., Obata A., Kikuchi M., Terao J. (2002). Unique uptake and transport of isoflavone aglycones by human intestinal caco-2 cells: Comparison of isoflavonoids and flavonoids. J. Nutr..

[27] Saeki T., Umezawa H., Tokieda-Fujishige T., Hori M. (1974). Letter: Biological effects of azomycin (2-nitro-imidazole): Inhibition of ribonucleotide reductase. J. Antibiot..

[28] Murphy M.P. (2009). How mitochondria produce reactive oxygen species. Biochem. J..

[29] Manca S., Magrelli A., Cialfi S., Lefort K., Ambra R., Alimandi M., Biolcati G., Uccelletti D., Palleschi C., Screpanti I. (2011). Oxidative stress activation of miR-125b is part of the molecular switch for Hailey-Hailey disease manifestation. Exp. Dermatol..

[30] Biolcati G., Aurizi C., Barbieri L., Cialfi S., Screpanti I., Talora C. (2014). Efficacy of the melanocortin analogue Nle4-d-Phe7-alpha-melanocyte-stimulating hormone in the treatment of patients with Hailey-Hailey disease. Clin. Exp. Dermatol..

[31] Kobayashi M., Yamamoto M. (2005). Molecular mechanisms activating the Nrf2-Keap1 pathway of antioxidant gene regulation. Antioxid. Redox Signal..

[32] Zhang D.D. (2006). Mechanistic studies of the Nrf2-Keap1 signaling pathway. Drug Metab. Rev..

[33] Tarasov A.I., Griffiths E.J., Rutter G.A. (2012). Regulation of ATP production by mitochondrial Ca^2+^. Cell Calcium.

[34] Kudla J., Becker D., Grill E., Hedrich R., Hippler M., Kummer U., Parniske M., Romeis T., Schumacher K. (2018). Advances and current challenges in calcium signaling. New Phytol..

[35] Missiaen L., Dode L., Vanoevelen J., Raeymaekers L., Wuytack F. (2007). Calcium in the Golgi apparatus. Cell Calcium.

[36] Missiaen L., Raeymaekers L., Dode L., Vanoevelen J., Van Baelen K., Parys J.B., Callewaert G., De Smedt H., Segaert S., Wuytack F. (2004). SPCA1 pumps and Hailey-Hailey disease. Biochem. Biophys. Res. Commun..

[37] Shull G.E., Miller M.L., Prasad V. (2011). Secretory pathway stress responses as possible mechanisms of disease involving Golgi Ca^2+^ pump dysfunction. BioFactors.

[38] Szigeti R., Kellermayer R. (2015). The forgotten yeast model of Hailey-Hailey disease. Int. J. Dermatol..

[39] Guthrie C., Fink G.R. (2002). Guide to Yeast Genetics and Molecular and Cell Biology, Part B.

[40] Cialfi S., Palermo R., Manca S., De Blasio C., Vargas Romero P., Checquolo S., Bellavia D., Uccelletti D., Saliola M., D’Alessandro A. (2014). Loss of Notch1-dependent p21(Waf1/Cip1) expression influences the Notch1 outcome in tumorigenesis. Cell Cycle.

