# Supplementary material for: Yeast-Based Screen to Identify Natural Compounds with a Potential Therapeutic Effect in Hailey-Hailey Disease
Source: Int J Mol Sci. 2018 Jun 20;19(6):1814. doi: 10.3390/ijms19061814 (PMC6032253; doi:10.3390/ijms19061814)
Supplement: Supplementary file 1 [file ijms-19-01814-s001.pdf]

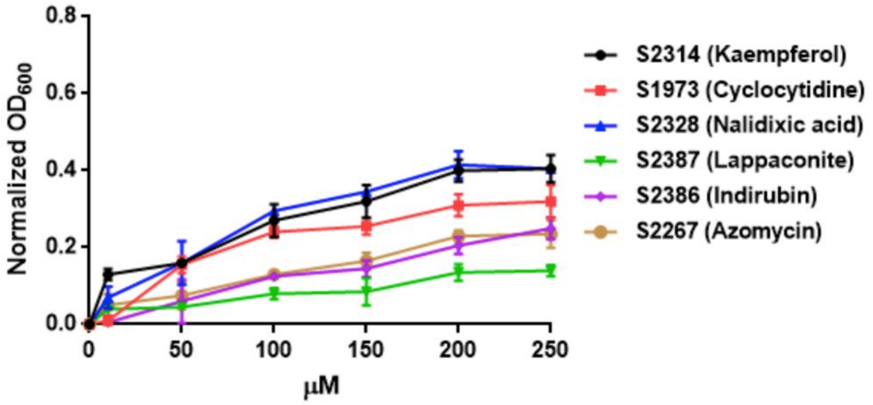

Figure S1. Dose-response curves of the six positive hits. *klpmr1Δ* cells were grown for 24h in the presence of different concentrations of the indicated compounds, then cells were challenged for further 24h with either menadione or H2O2, depending on the ability of each compound to allow the growth of *klpmr1Δ* cells using enadione or H2O2 as selective agents, as reported in Figure1. For Kaempferol only the dose-curve response in the presence of H2O2 is shown since its effect on cell growth in H2O2 was much stronger than that observed in menadione treated cells Figure1 The optical density was determined and normalized to OD600 of untreated cells.

Table S1. The collection of 131 natural compounds utilized in the screening. Asterisks indicate molecules that resulted toxic.

**S1973**	Cyclocytidine HCl	**S2314**	Kaempferol	**S2370**	Ursolic acid (Malol)
**S2250**	(-)-Epigallocatechin gallate	**S2316**	Kinetin (6-Furfuryladenine)	**S2371**	Vanillylacetone
**S2252**	(+)-Usniacin (D-Usnic acid)	**S2317**	L (+)-Rhamnose Monohydrate	**S2372**	Xanthone (Genicide)
**S2253**	3-Indolebutyric acid (IBA)	**S2319**	Limonin	**S2373**	Yohimbine hydrochloride (Antagonil)
**S2256**	4-Methylumbelliferone (4-MU)	**S2320**	Luteolin	**S2374**	5-hydroxytryptophan (5-HTP)
**S2258**	Aesculin (Esculin)	**S2321**	*Magnolol	**S2375**	Aloin (Barbaloin)
**S2260**	*Amygdalin	**S2322**	Matrine ((+)-Matrine)	**S2376**	Ammonium Glycyrrhizinate (AMGZ)
**S2261**	Andrographolide	**S2323**	Methyl-Hesperidin	**S2377**	*Biochanin A (4-Methylgenistein)
**S2262**	Apigenin	**S2325**	Morin hydrate (Aurantica)	**S2378**	Butylscopolamine bromide
**S2263**	Arbutin (Uva, p-Arbutin)	**S2326**	Myricetin (Cannabiscetin)	**S2379**	Dioscin (Collettiside III)
**S2265**	Artesunate	**S2327**	Myricitrin (Myricitrine)	**S2380**	Diosmetin (Luteolin 4-methyl ether)
**S2266**	Asiatic acid	**S2328**	Nalidixic acid (NegGram)	**S2381**	D-Mannitol (Osmitrol)
**S2267**	Azomycin (2-Nitroimidazole)	**S2329**	Naringin (Naringoside)	**S2383**	Gastrodin (Gastrodine)
**S2268**	*Baicalein	**S2331**	Neohesperidin dihydrochalcone (Nhdc)	**S2384**	Hematoxylin (Hydroxybrazilin)
**S2269**	Baicalin	**S2332**	Neohesperidin	**S2385**	Hordenine
**S2270**	Bergenin (Cuscutin)	**S2333**	Nobiletin (Hexamethoxyflavone)	**S2386**	Indirubin
**S2271**	*Berberine Hydrochloride	**S2334**	Oleanolic Acid (Caryophyllin)	**S2387**	Lappaconite Hydrobromide
**S2273**	β-Sitosterol	**S2335**	Oridonin (Isodonol)	**S2389**	Naringin Dihydrochalcone (Naringin DC)
**S2276**	Bilobalide	**S2336**	Orotic acid (6-Carboxyuracil)	**S2390**	Polydatin(Piceid)
**S2277**	Caffeic acid	**S2337**	Osthole (Osthol)	**S2391**	Quercetin (Sophoretin)
**S2280**	Chlorogenic acid	**S2338**	Oxymatrine (Matrine N-oxide)	**S2392**	Sesamin (Fagarol)
**S2281**	*Chrysin	**S2339**	Paeonol (Peonol)	**S2393**	Sorbitol (Glucitol)
**S2282**	Cinchonidine	**S2341**	*Parthenolide ((-)-Parthenolide)	**S2394**	Naringenin
**S2285**	Cryptotanshinone	**S2342**	Phloretin (Dihydronaringenin)	**S2395**	Rheochrysidin (Physcione)
**S2286**	Cyclosporin A (Cyclosporine A)	**S2343**	Phlorizin (Phloridzin)	**S2396**	Salidroside (Rhodioloside)
**S2287**	Cytisine (Baphitoxine, Sophorine)	**S2344**	Piperine (1-Piperoylpiperidine)	**S2397**	Palmatine chloride
**S2290**	Dihydroartemisinin (DHA)	**S2346**	Puerarin (Kakonein)	**S2399**	Dihydromyricetin (Ampeloptin)

**S2292**	Diosmin	**S2347**	Quercetin dihydrate (Sophoretin)	**S2401**	Sodium Danshensu
**S2293**	DL-Carnitine hydrochloride	**S2349**	Rutaecarpine (Rutecarpine)	**S2403**	Tetrandrine (Fanchinine)
**S2295**	Emodin	**S2350**	Rutin (Rutoside)	**S2404**	*Isoliquiritigenin
**S2296**	Enoxolone (Glycyrrhetin)	**S2351**	Salicin (Salicoside, Salicine)	**S2405**	Sophocarpine
**S2298**	Fisetin (Fustel)	**S2354**	Sclareol	**S2406**	Chrysophanic acid (Chrysophanol)
**S2299**	Formononetin (Formononetol)	**S2355**	Sclareolide (Norambreinolide)	**S2407**	Curcumol
**S2300**	Fumalic acid (Ferulic acid)	**S2356**	Shikimic acid (Shikimate)	**S2415**	Astragaloside A
**S2302**	Glycyrrhizic acid	**S2357**	Silibinin (Silybin)	**S2422**	Ipriflavone
**S2303**	Gossypol	**S2358**	Silymarin (Silybin B)	**S2423**	10-Hydroxycamptothecin
**S2304**	Gramine	**S2359**	Sinomenine (Cucoline)	**S2424**	Hypoxanthine
**S2306**	Gynostemma Extract	**S2362**	Synephrine (Oxedrine)	**S2425**	Apocynin (Acetovanillone)
**S2308**	Hesperetin	**S2363**	Tangeretin (Tangeritin)	**S2437**	Rotundine
**S2309**	Hesperidin	**S2364**	Tanshinone I	**S2439**	Guanosine
**S2310**	*Honokiol	**S2365**	Tanshinone IIA (Tanshinone B)	**S2442**	Inosine
**S2311**	Hyodeoxycholic acid (HDCA)	**S2366**	Taxifolin (Dihydroquercetin)	**S3071**	Vanillin
**S2312**	Icariin	**S2367**	Tetrahydropapaverine hydrochloride	**S3604**	Triptolide
**S2313**	Indole-3-carbinol	**S2369**	Troxerutin		

Table S2. Summary of the *klpmr1*Δ phenotypes analyzed to screen the natural product library.

**Compounds**	**menadione**	**H_2O_2**	**EGTA**	**CFW**	**DASPMI**
**S2387 (Lappaconite)**	++	-	-	++	++
**S2267 (Azomycin)**	++	+	-	++	-
**S2386 (Indirubin)**	+	-	+ +	++	++
**S2328 (Nalidixic acid)**	+	-	-	+++	-
**S1973 (Cyclocytidine)**	-	++	++	++	-
**S2314 (Kaempferol)**	+	++	-	++	+++

*(+++) = Totaly o strongly recovered; (++) = partialy recovered; (+) = slightly recovered; (-) = not recovered
